# Plasma membrane damage caused by listeriolysin O is not repaired through endocytosis of the membrane pore

**DOI:** 10.1242/bio.035287

**Published:** 2018-09-25

**Authors:** Lars Nygård Skalman, Mikkel R. Holst, Elin Larsson, Richard Lundmark

**Affiliations:** 1 Integrative Medical Biology, Umeå University, 901 87 Umeå, Sweden; 2 Medical Biochemistry and Biophysics, Laboratory for Molecular Infection Medicine Sweden, Umeå University, 901 87 Umeå, Sweden

**Keywords:** Membrane pore, Repair, Membrane damage, LLO, Listeriolysin, Caveolae, Clathrin-mediated endocytosis, Clathrin-independent endocytosis, CLIC

## Abstract

Endocytic mechanisms have been suggested to be important for plasma membrane repair in response to pore-forming toxins such as listeriolysin O (LLO), which form membrane pores that disrupt cellular homeostasis. Yet, little is known about the specific role of distinct endocytic machineries in this process. Here, we have addressed the importance of key endocytic pathways and developed reporter systems for real-time imaging of the endocytic response to LLO pore formation. We found that loss of clathrin-independent endocytic pathways negatively influenced the efficiency of membrane repair. However, we did not detect any increased activity of these pathways, or co-localisation with the toxin or markers of membrane repair, suggesting that they were not directly involved in removal of LLO pores from the plasma membrane. In fact, markers of clathrin-independent carriers (CLICs) were rapidly disassembled in the acute phase of membrane damage due to Ca^2+^ influx, followed by a reassembly about 2 min after pore formation. We propose that these endocytic mechanisms might influence membrane repair by regulating the plasma membrane composition and tension, but not via direct internalisation of LLO pores.

## Abstract (precis)

**Summary:** Using reporter systems and real-time imaging, we show that endocytic pathways are not directly involved in removal of LLO pores from the plasma membrane.

## INTRODUCTION

Plasma membrane integrity is critical for cellular homeostasis, and wounds in the plasma membrane need to be sealed rapidly to avoid cell death. For this purpose, cells have developed elaborate membrane repair mechanisms that are triggered by the influx of extracellular Ca^2+^ into the cytosol. The influx of Ca^2+^ induces a cascade of events including recruitment of membrane repair proteins to the site of damage and sealing of the damaged membrane in a matter of seconds (Andrews et al., 2014; Babiychuk and Draeger, 2015; Cooper and McNeil, 2015; Jimenez and Perez, 2015).

The mechanism for how the plasma membrane is repaired is contested and the current models include (i) patching using intracellular vesicles, (ii) microvesicle shedding, and (iii) endocytosis of the damaged membrane (Cooper and McNeil, 2015; Corrotte et al., 2013; Davenport et al., 2016; Jimenez et al., 2014; Keyel et al., 2011). The way that the wound is repaired is likely dependent on the nature of the wound, such as its size and if it was caused by mechanical rupture or pore-forming proteins (Cooper and McNeil, 2015; Jimenez and Perez, 2015). Mechanical ruptures, which leave a wound surrounded by lipids, could spontaneously reseal after a decrease in membrane tension as in the case of small wounds, or through endocytosis or shedding of the damaged membrane. Alternatively, the wound could be patched with fusing intracellular vesicles (Cooper and McNeil, 2015; Jimenez and Perez, 2015). However, pore-forming toxins (PFTs) that belong to the group of cholesterol-dependent cytolysins form large stable protein-lined pores in the plasma membrane. To repair this type of damage, the protein pore needs to be removed from the cellular membrane. PFT-induced plasma membrane damage is therefore believed to be repaired either via shedding of pore-containing microvesicles or by endocytosis of the pore (Andrews et al., 2014).

The role of endocytosis in membrane repair has gained increased attention during the last decade (Corrotte et al., 2013; Idone et al., 2008; Romero et al., 2017; Tam et al., 2010). It has been shown that influx of Ca^2+^ after plasma membrane damage caused by streptolysin O (SLO) toxin lead to lysosomal exocytosis and secretion of acid sphingomyelinase. This was proposed to promote ceramide formation in the outer leaflet of the plasma membrane and subsequent caveolar endocytosis of the pore (Corrotte et al., 2013; Tam et al., 2010). This model was strengthened by the observation that caveolin deficient cells were unable to repair SLO induced pores, and that the SLO toxin was seen to co-localise and be taken up via caveolae followed by lysosomal degradation (Corrotte et al., 2013, 2012). However, it has not been shown that caveolae directly internalise the active toxin pore. A number of questions regarding the role of caveolae and endocytic mechanisms for PFT removal are still to be answered. First, how are the pores/sites of damage specifically recognised by endocytic machineries and sorted into endocytic carriers? Second, are PFTs specifically internalised or do they trigger a generally increased rate of endocytosis? If so, what would be the mechanism that triggers endocytosis? Third, considering that endocytosis of an open pore could lead to leaky endosomes, would it potentially be a bad strategy to internalise the pore? Other proposed roles for endocytic mechanisms during membrane repair include restoration of the plasma membrane shape and composition back to normal after injury. Furthermore, endocytic proteins have been shown to promote the recruitment of membrane repair proteins to the wound site (Cooper and McNeil, 2015; Lenhart et al., 2015).

Here, we have studied the role that individual endocytic proteins play for efficient repair following membrane damage by the PFT, listeriolysin O (LLO). We have focused the studies on clathrin-mediated endocytosis, caveolae and clathrin-independent carriers (CLICs) due to their high prevalence and previously proposed role in membrane repair (Corrotte et al., 2013; Demonbreun et al., 2016a; Howes et al., 2010b). Live-cell microscopy of fluorescently tagged endocytic proteins together with annexin A6 was used to determine the correlation between pore formation and assembly of endocytic components. Our results show that proteins involved in clathrin-independent endocytic pathways influenced the membrane repair process. Surprisingly, we did not find any direct correlation between the endocytic proteins and the site of damage and no LLO internalisation by these pathways. Instead we propose that these different endocytic proteins are involved in the extensive reorganisation of the plasma membrane following this process.

## RESULTS

### Endocytic proteins influence the efficiency of membrane repair following LLO-induced plasma membrane damage

**Fig. 1.**
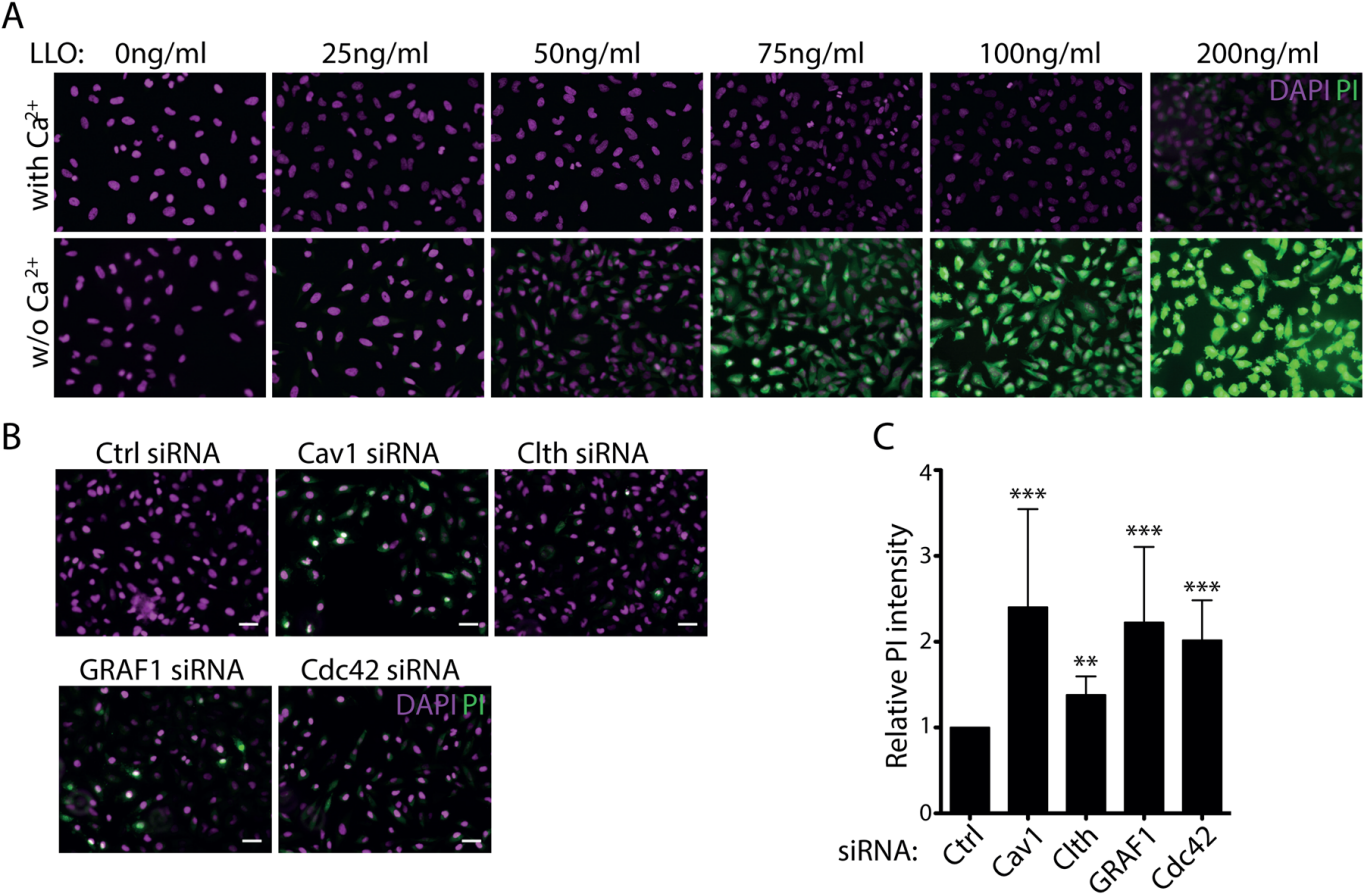
**Endocytic proteins are important for membrane repair of LLO induced damage of the plasma membrane.** (A) Representative fluorescent micrographs of HeLa cells treated for 5 min with different concentrations (as indicated) of LLO in medium with 1.8 mM Ca^2+^ (top) or with 5 mM EGTA (bottom). After LLO treatment the cells were incubated with PI, washed, fixed, stained with DAPI and imaged. (B) Representative fluorescent micrographs of HeLa cells treated with siRNA as indicated, exposed to media containing 1.8 mM CaCl_2_ with 75 ng/ml LLO (+LLO) or without LLO (control) for 5 min at 37°C, stained with DAPI and PI. (C) Bar plot of the mean PI intensity from fluorescent micrographs in the siRNA treated cells as indicated after treatment with LLO toxin. Data is representative of a minimum of three independent experiments. Mean relative PI intensity values: caveolin1=2.40, clathrin=1.38, GRAF1=2.23, cdc42=2.02. *P*-values of Mann–Whitney test compared to siRNA ctrl: caveolin1=0.0001, clathrin=0.003, GRAF1=0.0001, cdc42=0.0001. *n*=3. Error bars represent s.d. Scale bars: 25 μm.

To study membrane repair following damage from the PFT, LLO, we made use of a propidium iodide (PI) assay to monitor the permeability of the plasma membrane. When pores are formed in the cell surface, the fluorescent PI leaks into the cells and binds to DNA and RNA leading to a 20–30-fold increase in fluorescence that can be visualised by microscopy. Titration of the toxin concentration showed that HeLa cells could repair the damage following addition of 50–100 ng/ml of purified LLO when Ca^2+^ was present in the medium, but not when Ca^2+^ was depleted using EGTA (Fig. 1A). This showed that Ca^2+^ influx triggered active repair of the membrane in HeLa cells at this toxin concentration. To test the importance of different endocytic machineries in membrane repair, we depleted cells of key proteins known to affect clathrin-dependent uptake (clathrin), clathrin-independent carriers (CLIC) uptake (GRAF1, cdc42) or caveolae (caveolin1) using siRNA. The efficiency of siRNA-based depletion was verified by immunoblotting (Fig. S1A). Cells were then treated with 75 ng/ml LLO and analysed using the PI assay. Compared to control cells, the removal of GRAF1, cdc42 or caveolin1 had a large effect on PI uptake (Fig. 1B). GRAF1 and cdc42 are known to affect CLIC endocytosis and the organisation of cortical actin (Doherty and McMahon, 2009), while caveolin1 is integral for the formation of caveolae (Parton and del Pozo, 2013). The effect of clathrin HC depletion, and thereby clathrin-dependent endocytosis was not as prominent*.*

### Annexin A6 marks LLO-induced plasma membrane damage in HeLa cells

**Fig. 2.**
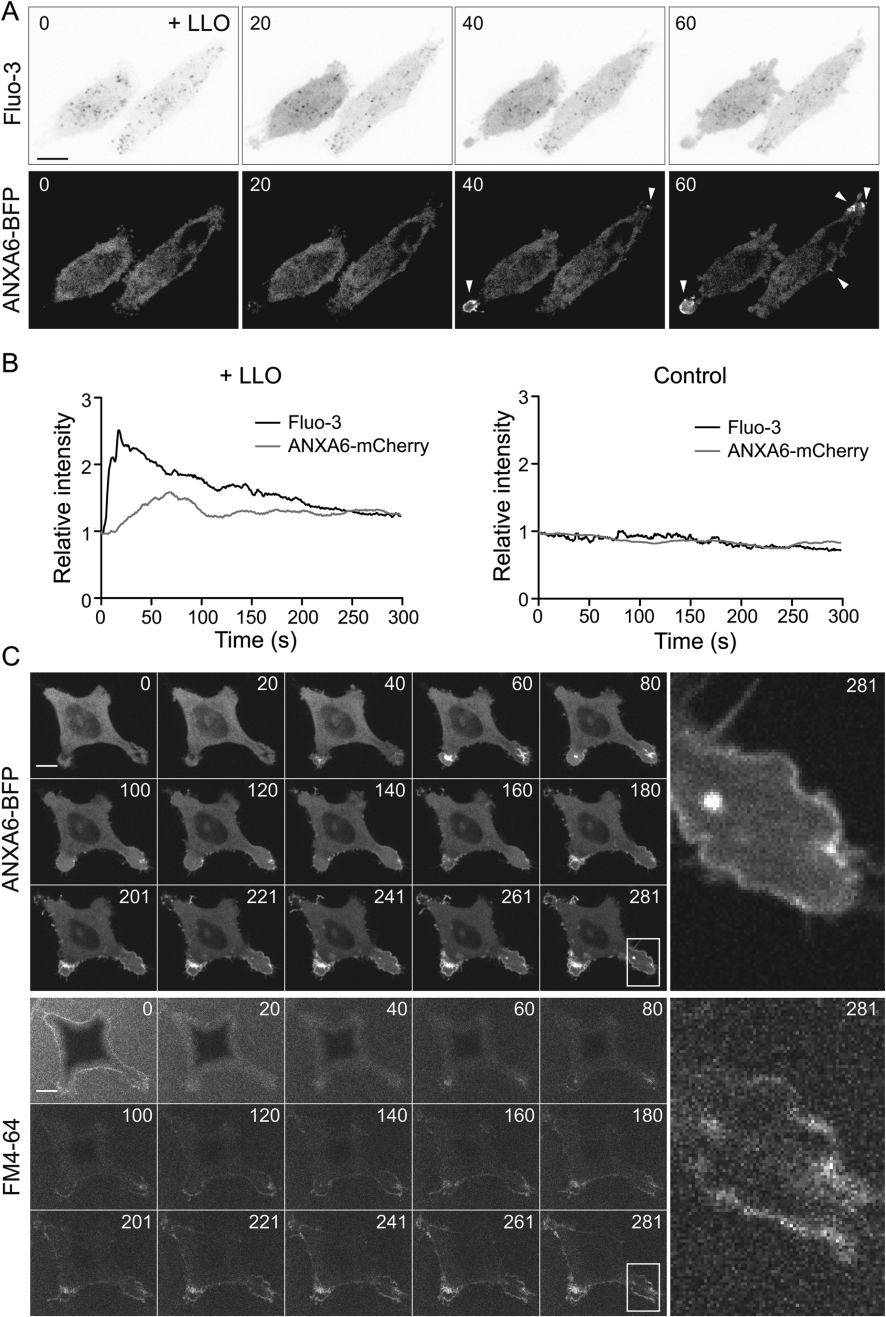
**Annexin A6 is recruited to the site of LLO induced damage after an increase in cytosolic Ca^2+^.** (A) Live-cell confocal spinning disc time series of Fluo-3 and ANXA6-mCherry taken at 1 fps showing an increase in cytosolic Fluo-3 intensity (inverted top panel) and annexin A6 recruitment to the plasma membrane (white arrow head bottom panel) following LLO-treatment. (B) Graphs showing the quantification of the relative intensity of cytosolic Fluo-3 and specific ANXA6-mCherry in a ROI at a plasma membrane protrusion in LLO treated and control cells. The mean intensity of each channel in the first frame of the movie was set as 1. Time 0 represent the acquisition start time in all subfigures. Time in seconds. (C) Live-cell confocal spinning disc time series of ANXA6-BFP transfected HeLa Flp-In TRex cells treated with LLO and FM4-64. Time series of 281 s of ANXA6-BFP (upper three rows) and FM4-64 (lower three rows) are presented. Insets to the right, show magnification of the area indicated by a white square and illustrates that FM4-64 and Annexin A6 stainings are similar. Time is indicated in seconds. Scale bars: 10 μm.

To study the acute endocytic response specific to LLO-pore formation we set up a live cell imaging assay based on annexins as probes of pore formation. Annexins, a family of Ca^2+^ and phospholipid binding proteins, have been shown to respond to Ca^2+^ influx by binding to the plasma membrane resulting in assembly of annexins at membrane pores and sites of damage (Babiychuk and Draeger, 2015; Boye et al., 2017; Demonbreun et al., 2016a). To test if pore-formation by LLO also triggered this annexin response we co-transfected ANXA6-mCherry with the Ca^2+^ probe Fluo-3. Live cell imaging revealed that LLO-treatment caused an almost immediate increase in the general cytoplasmic Fluo-3 fluorescence revealing Ca^2+^ influx, which was followed by a subsequent surface assembly of ANXA6-mCherry (Fig. 2A). Analysis of the fluorescent intensity revealed that the annexin A6 response appeared approximately 10–40 s after the increase in Fluo-3 fluorescence (Fig. 2B). Without toxin addition, no response in the cytoplasmic fluorescent signal of Fluo-3 nor annexin A6 was observed.

To verify that annexin A6 was recruited to sites of membrane damage, we used FM4-64 to label membrane lesions. FM4-64, a lipophilic dye that display increased fluorescence following binding to negatively charged lipids exposed upon membrane damage (Demonbreun et al., 2016a). FM4-64 was added to cells transiently transfected with BFP-tagged annexin A6 (ANXA6-BFP). When cells were treated with LLO, ANXA6-BFP started to assemble in bright structures of varying shapes and sizes at the cell surface within 40 s following toxin addition (Fig. 2C). Notably, FM4-64 accumulated in membrane areas where ANXA6-BFP assembled, confirming that this was sites of membrane damage (Fig. 2C). No change in the fluorescent signal of FM4-64 nor annexin A6 was detected without toxin addition (Fig. S2). These results show that fluorescently tagged ANXA6 could be used to mark membrane lesion caused by LLO addition to cells. As previously observed, annexin A6 assemblies induced by toxin addition varied in their morphology ranging from small spots at the surface, protruding spikes and assemblies covering larger surface areas (Fig. S3). While the larger areas of annexin A6 were transient, the small spots and protruding spikes often persisted over the entire 5 min of which the movie was recorded. All these types of structures were regarded as an indication of membrane damage (Fig. S3).

### LLO-induced membrane damages do not trigger endocytic removal

**Fig. 3.**
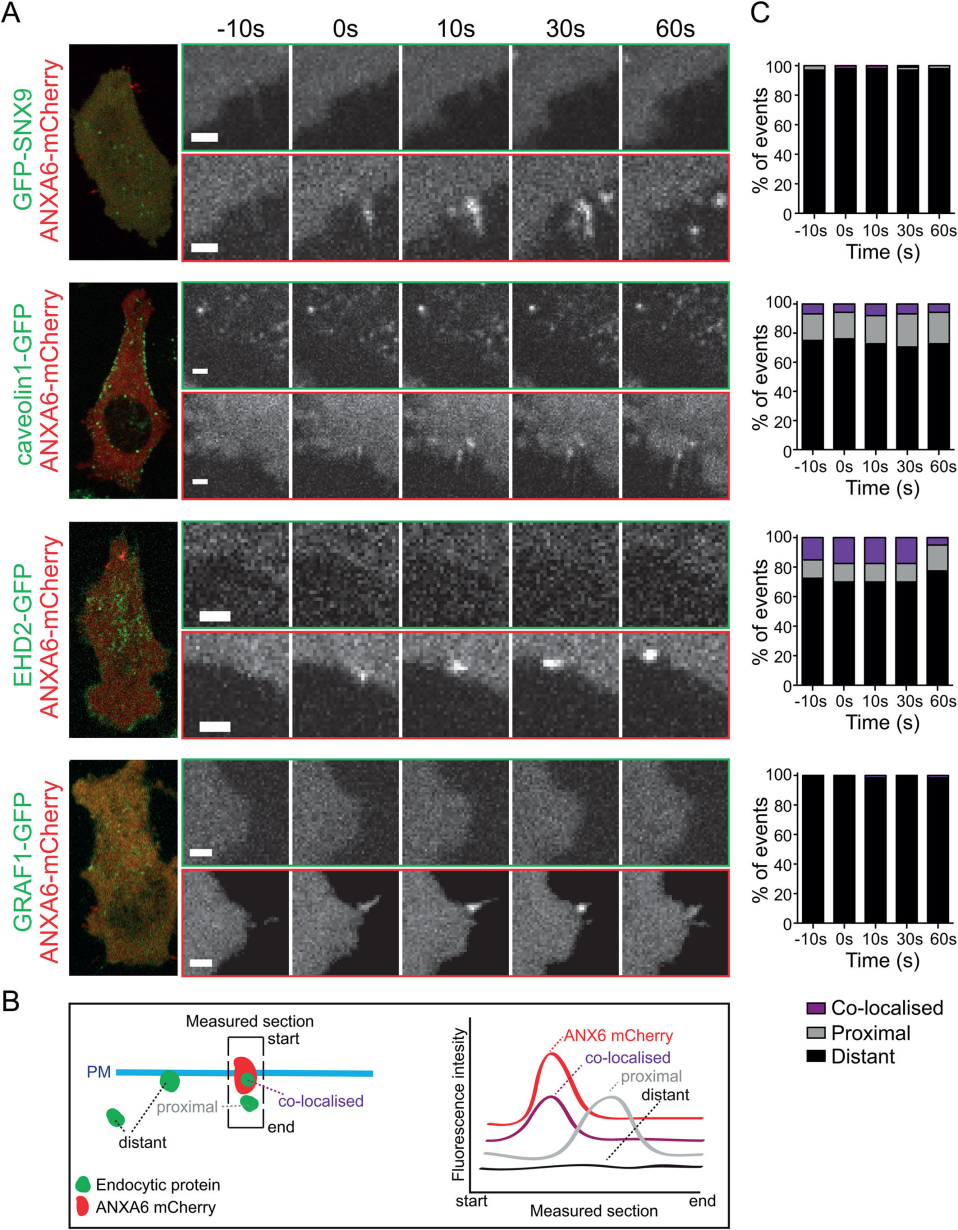
**Endocytic proteins are not recruited to sites of annexin A6 following LLO addition.** (A) Representative images from live-cell confocal spinning disc imaging of ANXA6-mCherry structures and localisation of endocytic proteins to these sites at different time points. To the left is an overview of the cell at time point 0. Movies were taken at 1 fps, time is shown in seconds. Scale bars: 2 μm. Live-cell confocal spinning disc microscopy of GFP-SNX9, caveolin1-GFP, EHD2-GFP and GRAF1-GFP Flp-In TRex cells transfected with ANXA6-mCherry and treated with LLO. (B) Schematic illustration of the plasma membrane (PM) and annexin and endocytic assemblies indicating how the section for measuring intensity of the two channels was drawn, and how the fluorescence intensity was measured and categorised. The section was drawn towards all visible endocytic structures at or in the proximity of annexin A6 structures. The localisation of endocytic proteins to assemblies of ANXA6-mCherry was quantified as; co-localised, proximal (if within 1 μm), and as distant if no correlation was found by analysing the intensity of both GFP and mCherry using a line drawn over the ANXA6-mCherry structures. (C) Quantifications of the localisation of endocytic proteins to the ANXA6-mCherry structures 10 s before the appearance of ANXA6-mCherry (−10 s), at the time of recruitment (0 s) and 10, 30 and 60 s after recruitment. 40–100 events in total were quantified for each endocytic protein.

Having established an assay for spatial and temporal visualisation of LLO pore formation, we aimed to study if the key endocytic proteins observed to negatively impact membrane repair after LLO-treatment (Fig. 1) were involved in direct endocytosis of the pores. To visualise the endocytic proteins, we used HeLa Flp-In TRex cell lines stably expressing the GFP-tagged proteins caveolin1, EHD2, GRAF1 or SNX9, respectively, following addition of doxycycline. These cell lines enable controlled protein expression levels in the endogenous range and have been previously characterised (Daste et al., 2017; Francis et al., 2015; Hoernke et al., 2017; Mohan et al., 2015). Caveolin1 is required for the formation of caveolae and EHD2 confines caveolae to the cell surface, while GRAF1 and cdc42 are known to affect CLIC endocytosis and the organisation of cortical actin (Mayor et al., 2014). SNX9 participates in the late events of clathrin coated vesicle formation (Daste et al., 2017). To mark the pores ANXA6-mCherry was transiently transfected into the HeLa Flp-In TRex cell lines and using live-cell spinning disc confocal microscopy, cells were imaged before and after addition of LLO (Fig. 3A; Movies 1–4).

In order to analyse the degree of spatial correlation between the endocytic proteins and the site of annexin A6 assembly we categorised the localisation of the fluorescent assemblies of the individual endocytic markers as co-localised, proximal or distant to annexin A6. This was done by measuring the fluorescence intensity of the endocytic proteins within a section spanning the membrane ranging from the extracellular space in to the cytosol covering the annexin A6 structure (Fig. 3B, illustration). The peak intensities of both channels were measured at time of the appearance of ANXA6-mCherry (0 s) as well as 10 s before and 10, 30 and 60 s after appearance (Fig. 3A). An intensity peak at the same site in both channels was interpreted as co-localisation, peaks within 1 μm was categorised as proximal and no correlation as distant. Surprisingly, we found no significant co-localisation between any of the endocytic markers and annexin A6 assemblies at any of the time points (Fig. 3C; Movies 1–4). The percentages of proximal and co-localised spots were slightly higher for caveolin1 and EHD2, likely due to their abundant and stable localisation at the surface as compared to SNX9 and GRAF1. However, no time dependent increase in their co-localisation was detected. These data showed that markers of clathrin-dependent endocytosis, clathrin-independent endocytosis and caveolae are not recruited to membrane damage sites marked by annexin A6, suggesting that such sites are not internalised by these pathways.

### LLO is sequestered at the cell surface and not specifically endocytosed

**Fig. 4.**
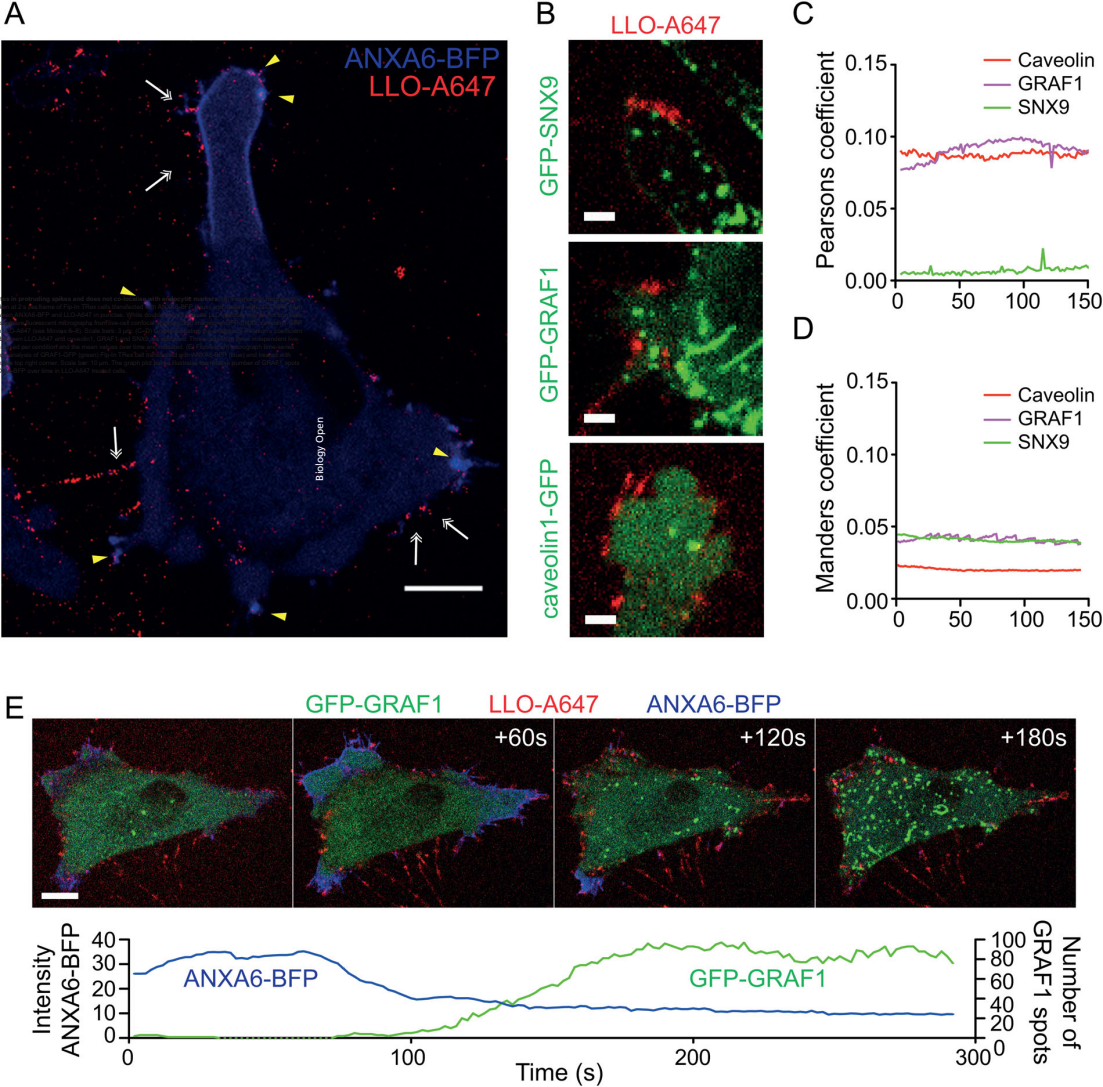
**Fluorescently labelled LLO accumulates** **in protruding spikes and does not co-localise with endocytic markers.** (A) Fluorescent micrographs from live-cell confocal spinning disc imaging taken at 2 s per frame of Flp-In TRex cells transfected with ANXA6-BFP (blue) and treated with LLO-A647 (red). Yellow arrowheads indicate co-localization between ANXA6-BFP and LLO-A647 in punctae. White double arrows indicate LLO-punctae that do not correlate with ANXA6-BFP. Scale bar: 10 μm. (B) Representative fluorescent micrographs from live-cell confocal spinning disc imaging of GFP-SNX9, caveolin1-GFP and GRAF1-GFP Flp-In TRex cells treated with LLO-A647 (see Movies 6–8). Scale bars: 3 μm. (C–D) Graph illustrating the analysis of Pearson’s coefficient (C) and Manders coefficient and (D) over time between LLO-A647 and caveolin1, GRAF1 and SNX9, as indicated. Three cells from three independent live-cell experiments taken at 2 s per frame were analysed per condition and the mean values over time are indicated. (E) Fluorescent micrograph time-series from a live-cell confocal spinning disc microscopy analysis of GRAF1-GFP (green) Flp-In TRex cell transfected with ANXA6-BFP (blue) and treated with LLO-A647 (red). Time in seconds is indicated in the top right corner. Scale bar: 10 μm. The graph plot below illustrates the relative number of GRAF1 spots and the relative intensity (Arbitrary units) of ANXA6-BFP over time in LLO-A647 treated cells.

To directly track the LLO toxin and test if we could detect endocytosis of LLO independent of whether it induced pores and an annexin response or not, we used fluorescently labelled LLO-A647. Following addition to cells, binding of LLO-A647 was detected in discrete punctae and protruding spikes over the entire cell surface (Fig. 4A; Movie 5). Most of these persisted throughout the 5 min movies. A clear annexin A6 response following addition of the labelled toxin to cells verified that it generated pores at the same concentration as unlabelled toxin (Fig. 4A; Fig. S4). Many of the LLO-A647-punctae co-localised with specific annexin A6-positive assemblies verifying annexin A6 can be used to mark sites of LLO-pores (Fig. 4). We also observed LLO-A647-punctae negative for an annexin A6 response, indicating that not all of the labelled toxin caused membrane pores.

To test if endocytic proteins were co-localising with the labelled toxin, Flp-In cells expressing either GFP-tagged GRAF1, caveolin1 or SNX9 were monitored by live-cell microscopy following addition of LLO-A647 (Fig. 4B). Binding of the labelled toxin to the cell surface in discrete punctae over time was similar in between these cell lines and control cells (Movies 5–8). However, we did not observe any specific enrichment of LLO in any of the assemblies of caveolin1, GRAF1 or SNX9 (Fig. 4B; Movies 6–8). Analysis of the co-localisation between LLO and the different endocytic marker proteins over the 5 min movies revealed low levels of co-occurrence (co-localisation) (Fig. 4C) and weak correlation of the intensities (Fig. 4D). We did not detect any enrichment of LLO within the cells over time, suggesting that most of the LLO was sequestered at the cell surface or shed from the cells (Movies 6–8). This suggested that LLO was not specifically targeted to any of these endocytic sites or internalised via these pathways. Interestingly however, we found that the number of GRAF1-assemblies seemed to inversely correlate with the annexin A6 response to LLO. During the annexin response, very few GRAF1 carriers were detected, but following loss of the annexin A6 signal, an extensive upregulation in the number of GRAF1-carriers was detected in a subset of cells (Fig. 4E; Movie 9). This suggested that although these pathways were not involved in specific uptake of LLO, the recovery from membrane damage influenced the activity of CLIC endocytosis.

### LLO-mediated influx of Ca^2+^ results in acute loss of GRAF1-positive assemblies at the cell surface

**Fig. 5.**
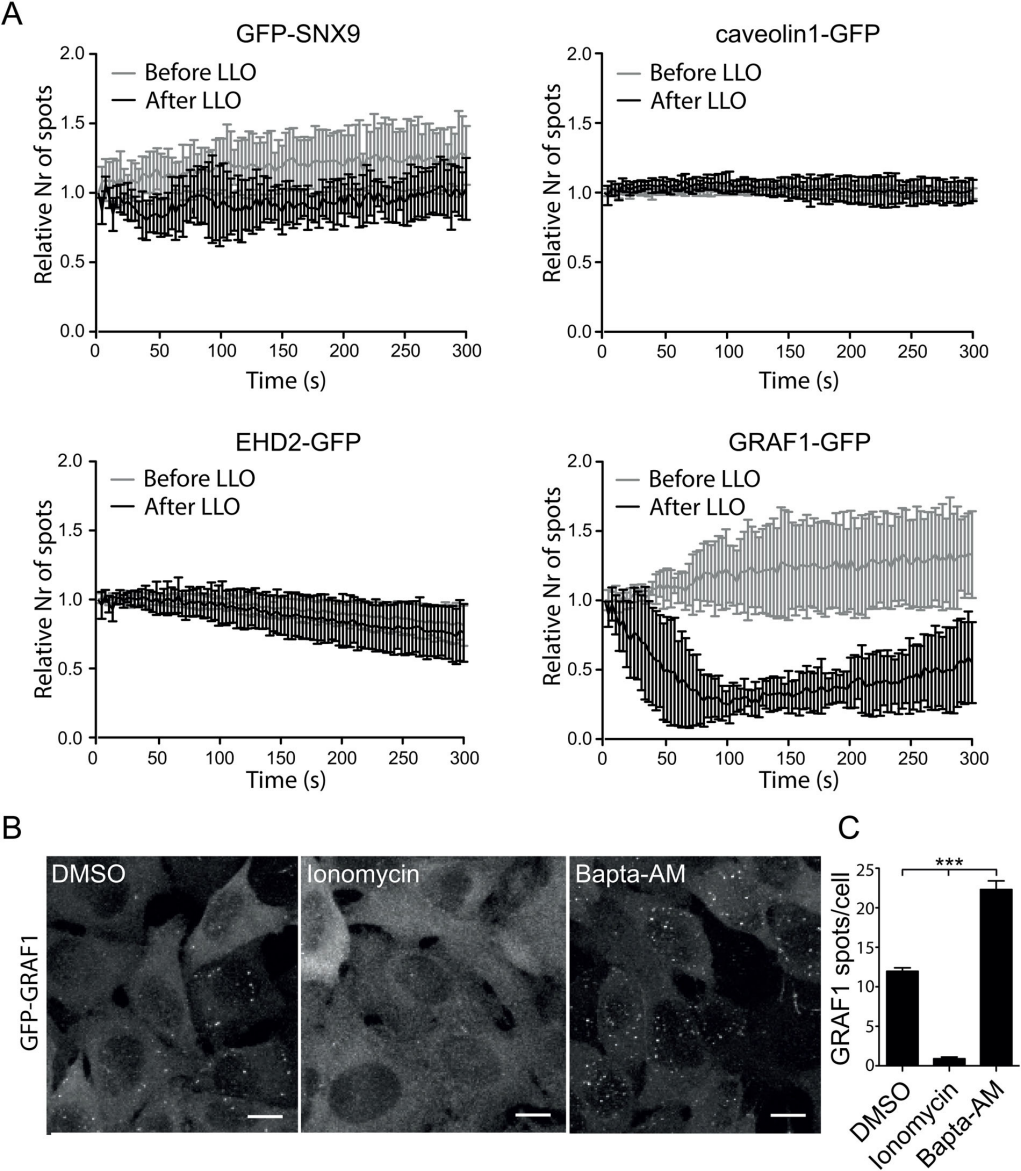
**Pore formation by LLO represses GRAF1 assembly but does not influence the number of caveolin1- or SNX9-positive structures.** (A) Graphs showing the number of endocytic spots detected by live-cell TIRF microscopy of HeLa Flp-In TRex cells expressing different endocytic proteins treated with LLO. The number of endocytic structures present in the TIRF field was quantified every 3 s for 5 min before and after LLO addition and plotted as the relative number of spots where the first frame of respective movie was set as 1. Error bars represent the s.d. Only movies with cells showing ANXA6-mCherry recruitment to the plasma membrane after addition of LLO were quantified. Time 0 s represent the acquisition start time in all subfigures. The data represent quantifications derived from several cells and independent movies from three independent experiments *n*=3 except for GRAF1, where *n*=1 on 7 cells. (B) Representative micrographs of maximum-projected confocal z-stacks showing fixed Flp-In TRex cells expressing GFP-GRAF1. Vehicle DMSO (control), 10 μM Ionomycin and 50 μM Bapta-AM was added to the cells for 30 min followed fixation and imaging, respectively, as indicated. Scale bars 10 μm. (C) Bar plot of the number of GFP-GRAF1 structures quantified after 30 min of drug treatment. *P*-values from one-way ANOVA: control versus Ionomycin *P*<0.01, control vs Bapta AM *P*<0.0001, Ionomycin vs Bapta AM *P*<0.0001. *n*=3. Error bars represent s.d.

Altered homeostasis and membrane integrity in response to membrane damage might influence the turnover of the plasma membrane. To test if the general activity of the endocytic machineries was affected by the treatment with LLO, the number of surface assemblies was measured over time. The spatial and temporal cell surface localisation of GFP-SNX9, caveolin1-GFP, EHD2-GFP or GRAF1-GFP in living cells were analysed by total internal reflective fluorescence (TIRF) microscopy. We imaged the surface assembly of these proteins before and after addition of LLO, and tracked the number of assemblies over time using imaging software (Fig. 5A). Cells were co-transfected with ANXA6-mCherry to verify membrane damage. No difference in the number of endocytic assemblies could be detected for caveolin1, EHD2 or SNX9 (Fig. 5A), in agreement with previous experiment. However, we found a dramatic reduction in the number of GRAF1-assemblies (Fig. 5A) confirming previous data. The decrease started immediately following toxin addition and after around 100 s we noticed that the number of GRAF1-assemblies started to increase again. The immediate decrease in GRAF1-assemblies suggested that the membrane association of GRAF1 might be sensitive to the intracellular Ca^2+^ levels. To address this, we treated GRAF1 Flp-In TRex cells with the drug Ionomycin, which elevate intracellular Ca^2+^ levels, or with Bapta-AM, which chelates intracellular Ca^2+^. Cells were fixed and GRAF1-assemblies were visualized by confocal microscopy (Fig. 5B). Quantification of the number of GRAF1-assemblies showed that Ionomycin treatment completely abolished GRAF1-assemblies, whereas Bapta-AM treatment doubled the amount of GRAF1-assemblies in cells, in comparison to control cells (Fig. 5C). This implied that the acute increase in Ca^2+^ following membrane damage blocks the assembly of GRAF1 at the cell surface, thereby inhibiting CLIC endocytosis.

Taken together, our results indicate that endocytic machineries contribute to the ability to endure treatment with LLO. However, the initial repair following pore-formation and annexin-recruitment does not involve internalisation by the endocytic mechanisms assayed here. Rather, endocytic machineries might be involved in readjusting the composition of the plasma membrane following damage.

## DISCUSSION

Endocytosis has been suggested to play an important role during membrane repair of PFT pores as well as other types of membrane damage. However, the precise role, and the spatial and temporal response of different endocytic pathways to PFT induced damage is unclear. Here, we characterised the importance and dynamics of different endocytic pathways in response to the LLO toxin. We found that depletion of caveolin1, GRAF1 and cdc42, which are known to impair clathrin-independent endocytic pathways, negatively influenced the ability of HeLa cells to repair after LLO-mediated damage. The effect of caveolin1 depletion is consistent with previous results based on membrane repair of SLO toxin, mechanical and laser induced wounds (Corrotte et al., 2013), suggesting that caveolae impacts on membrane repair. Also GRAF1, a marker for CLIC endocytosis (Lundmark et al., 2008), has previously been shown to be important for membrane repair in mouse skeletal and cardiac muscle cells after laser induced wounds (Lenhart et al., 2015; Lundmark et al., 2008). Together with our results, this would suggest that GRAF1 has a general role in membrane repair irrespective of cell type and the nature of the wound. In agreement with that CLIC endocytosis influence repair, depletion of the Rho GTPase cdc42 also affected the repair process. This could also reflect a more general effect on cell surface integrity due to the central role of cdc42 in cytoskeletal organisation (Croisé et al., 2014; de Curtis and Meldolesi, 2012). Cdc42 has also been shown to be involved in patching of laser induced wounds in *Xenopus laevis* oocytes (Davenport et al., 2016). Both caveolae and CLICs are proposed to work as membrane buffering systems, with the capacity to rapidly, but rather unspecifically, turn over membranes (Holst et al., 2017; Howes et al., 2010a; Sinha et al., 2011). Clathrin-mediated endocytosis, on the other hand, is responsible for uptake of specific receptors, and in line with previous studies, we did not find any major impairment of membrane repair following depletion of clathrin (Idone et al., 2008).

The acute endocytic response to membrane pore formation has been difficult to address. Using high-end live-cell microscopy and genetically designed marker cell lines, we have studied the potential endocytosis of both the pore and the toxin in great spatiotemporal detail. Pore formation was detected using annexin A6, which is one of the most Ca^2+^ sensitive annexins and has been shown to translocate to sites of membrane damage within a matter of seconds (Babiychuk and Draeger, 2015; Demonbreun et al., 2016b; Potez et al., 2011). We could confirm that this also applied to pores induced by LLO since ANXA6-BFP co-localised with the lipidic dye FM4-64 after LLO addition. Using the Ca^2+^ reporter Fluo-3 we found that annexin A6 was recruited approximately 10–40 s after Ca^2+^ influx in HeLa cells. In addition to labelling the site of damage, annexin A6 served as an internal control for toxin overload in cells. In cells unable to repair the LLO induced damage, annexin A6 labelled the whole plasma membrane as well as internal vesicles as previously shown (Babiychuk and Draeger, 2015). Surprisingly, we did not observe any specific recruitment of the marker proteins caveolin1, EHD2, GRAF1, SNX9 to the membrane damage sites labelled by annexin A6. Neither did we observe any increase in the surface assembly of any of the endocytic proteins following induction of membrane repair. This suggested that these endocytic pathways are not directly involved in pore removal as a means of membrane repair. Using a fluorescently labelled toxin, we found that most of the toxin was sequestered at the cell surface. Very little overlap between the toxin and any of the endocytic markers was observed, suggesting that, independent of pore formation, LLO is not specifically internalised by these mechanisms. Our results are in agreement with a recent paper where the authors show that pores created by other PFTs induce excessive shedding of the toxin packed in microvesicles (Romero et al., 2017). The fact that the ESCRT-machinery has been shown to recruit to LLO induced pores (Jimenez et al., 2014) further supports the notion that the LLO pores are shed from the plasma membrane. In our microscopy-based assay, both labelled LLO and the annexin A6 response to pore formation was enriched in thin protruding spikes. This is similar to what has been shown before with annexin A1 after treatment with the SLO toxin (Atanassoff et al., 2014). However, the potential shedding of microvesicles from these spikes is difficult to convincingly visualise using a microscopy-based assay since free vesicles rapidly move out of the focus plane.

CLICs are known as a high capacity mechanism for turnover of the plasma membrane in response to acute changes in membrane tension (Holst et al., 2017; Howes et al., 2010a; Vidal-Quadras et al., 2017). Notably, we found that the number of GRAF1 assemblies decreased rapidly after addition of LLO, and that GRAF1 assemblies started to reappear in many cells when the annexin A6 response decreased. The disassembly of GRAF1 was likely due to a rise in intracellular Ca^2+^ levels upon membrane damage, as an increase in cytoplasmic Ca^2+^ decreased the number of assemblies, while Ca^2+^ depletion did the reverse. This might indicate that during acute damage, Ca^2+^ is used as a signal to promote exocytosis and limit endocytosis so that membrane pools can be used for repair. Although depletion of GRAF1 hampered the cells ability to repair LLO pores, it is evident from our results that GRAF1 endocytosis is not involved in the initial repair of LLO pores. However, GRAF1 might have a role maintaining intracellular membrane pools that can be used for repair. Following damage and repair the membrane pools might need to be regenerated, which could explain the increased GRAF1 activity when the levels of Ca^2+^ have been restored to normal.

The fact that we did not detect any co-localisation between caveolin1 and the toxin, as well as finding no effect on caveolae dynamics is surprising since caveolin1 depletion made cells more susceptible to LLO treatment. Also, in a previous study where SLO toxin was used, it was found that caveolae endocytosis was stimulated after toxin addition (Corrotte et al., 2013). It is however possible that differences in experimental setup affects the obtained results. We added the LLO toxin at 37°C, while the SLO was bound on ice and then increased the temperature to 37°C in the other study (Corrotte et al., 2013). Low temperatures can significantly alter the properties of the plasma membrane and are known to affect certain endocytic pathways (Doherty and McMahon, 2009). Also, it has been proposed that the amount of damage determines the repair mechanism; while a low level of plasma membrane damage mainly induced shedding of toxin pores, excessive damage induced large internalisation events (Atanassoff et al., 2014). Furthermore, caveolae regulate the lipid homeostasis in the plasma membrane and function as mechano-sensitive membrane reservoirs (Ariotti et al., 2014; Gaus et al., 2006; Sinha et al., 2011) and it is likely that both of these features are important for an efficient membrane repair.

Endocytosis has been suggested to internalise the damaged membrane within seconds after injury following ceramide formation in the plasma membrane (Idone et al., 2008; Tam et al., 2010). However, the specificity of ceramide-induced endocytosis for pore removal should be considered because it would require abundant ceramide formation at the exact site of the wound in order to recruit endocytic mechanisms to this location. Repair of SLO induced pores has been shown to occur in lysosomal free protrusions, suggesting another type of repair mechanism at these sites (Atanassoff et al., 2014). Lysosomal exocytosis in response to membrane damage may significantly alter local surface charge of the plasma membrane and its curvature. An altered composition together with a change in membrane morphology and tension may require both caveolae and the high capacity CLIC pathway to be activated in order to restore plasma membrane integrity. Our data suggests that the buffering of membrane composition and tension might be an important aspect of the repair process following membrane pore formation.

In conclusion, we find that loss of clathrin-independent endocytic proteins negatively influences the ability of cells to recover from LLO-induced damage. However, LLO pores marked by annexin A6 recruitment are not internalised (within the analysed time frame) by any of the endocytic pathways studied in here. Our data supports that LLO is sequestered at the cell surface and that endocytic proteins rather might be involved in reorganisation of the cell surface in response to damage.

## MATERIALS AND METHODS

### Reagents and plasmids

Recombinant LLO toxin was from Diatheva and aliquots were stored in 50 mM NaH_2_PO_4_, 0.5 M NaCl, 2.7 mM KCl, 1 mM EDTA, 1 mM DTT and 5% (v/v) glycerol at −80°C. FM4-64FX and Fluo-3 were from Molecular Probes, Ionomycin from LC Laboratories and Bapta-AM from Tocris Bioscience. ANXA6-mCherry (pN1-A6-mCherry) was a kind gift from Anette Draeger (University of Bern, Switzerland). To generate the ANXA6-BFP vector, the ANXA6 gene was subcloned into a pTagBFP-N vector using the NheI and XhoI restriction sites. Recombinant LLO toxin was labelled with a tenfold excess of Alexa Fluor 647 with a NHS-ester (Invitrogen) in 50 mM NaH_2_PO_4_, 0.5 M NaCl, 2.7 mM KCl, 1 mM EDTA, 100 mM NaHCO_3_ for 30 min at RT. Unreacted dye was separated on an Illustra Nap-5 column (GE Healthcare Life Sciences) and aliquots were stored in 50 mM NaH_2_PO_4_, 0.5 M NaCl, 2.7 mM KCl, 1 mM EDTA, 1 mM DTT and 5% (v/v) glycerol at −80°C. Activity of LLO-A647 was confirmed by using the PI assay to be similar to unlabelled LLO toxin.

### Cell lines and transfection

HeLa cells (ATCC-CRM-CCL-2) were maintained in DMEM supplemented with 10% FBS. HeLa Flp-In TRex caveolin1 GFP (Mohan et al., 2015), GFP-SNX9 (Daste et al., 2017), GFP-GRAF1 and GRAF1-GFP (Francis et al., 2015) were generated before. EHD2-GFP was generated as previously described (Mohan et al., 2015; Tighe et al., 2008). The HeLa Flp-In TRex cell lines were grown in DMEM supplemented with 10% FBS, 100 μg/ml Hygromycin B and 5 μg/ml Blasticidin (InvivoGen). Cell lines were checked for mycoplasma infection. GFP expression in Flp-In TRex cells was induced by the addition of doxycycline hyclate (Sigma-Aldrich) to the cell culture medium 1 h before transfection with plasmid DNA. Caveolin1 GFP and EHD2-GFP were induced with 0.5 ng/ml doxycycline and GRAF1-GFP, GFP-GRAF1, GFP-SNX9 with 1 ng/ml doxycycline. Plasmid DNA was transfected into cells using Lipofectamine 2000 (Invitrogen) for 6 h at 20–24 h before the experiment.

### siRNA transfection and immunoblotting

HeLa cells were transfected with siRNA using Lipofectamine 2000 for 2×48 h or 1×72 h according to the manufactures’ recommendations. The siRNAs used were human-specific stealth siRNA (Invitrogen) for siRNA ctrl (negative control medium GC duplex), caveolin1 (HSS141467), Clathrin HC (HSS102017), GRAF1 (HSS118163) and cdc42 On target plus siRNA (J-005057-05) (Dharmacon). The cells were seeded on coverslips 24 h before optimal knockdown for PI assay. The efficiency of siRNA-based knockdown of endocytic proteins was determined by western blot. Primary antibodies used were mouse anti-GAPDH 1:5000 (MAB374, Millipore), rabbit anti-caveolin1 1:10,000 (ab2910, Abcam), mouse anti-Clathrin HC 1:1000 (clone 23 610499, BD Biosciences), rabbit anti-GRAF1 (Ra83) (Lundmark et al., 2008) and rabbit anti-cdc42 1:1000 (ab109553, Abcam). Secondary antibodies, donkey anti-mouse and donkey anti-rabbit IgG coupled to IRDye 680LT or 800CW (Li-Cor Biosciences) were used for fluorescent detection using an Odyssey Sa instrument (Li-Cor Biosciences). For detection with ECL Prime (GE Healthcare) anti-rabbit and anti-mouse IgG conjugated to horseradish peroxidase (HRP) from Agrisera or Sigma-Aldrich were used. The level of knockdown was quantified using Image Studio (Li-Cor Biosciences) for fluorescent images and ImageJ for films (Schneider et al., 2012).

### Propidium iodide assay

For titration of LLO concentrations 1×10^5^ HeLa cells were seeded on coverslips one day before the experiment. The cells were washed 1× in serum free DMEM containing 1.8 mM CaCl_2_ or in CaCl_2_ and serum free DMEM supplemented with 5 mM EGTA and subsequently incubated for 5 min at 37°C with or without LLO toxin. The cells were stained with 50 μg/ml propidium iodide (PI) in DMEM or in calcium free DMEM with 5 mM EGTA and washed in PBS 1× followed by fixation for 10 min in 3% PFA in PBS. After fixation, the cells were washed, permeabilised with 0.1% triton for 1 min and stained with DAPI. Images of PI stained cells were taken with an epifluorescence Zeiss Axio Imager Z1 microscope controlled by the Zen software (Zeiss, Germany) directly after staining. Essentially the same protocol was used for the PI assay on siRNA treated HeLa cells. Quantification of the mean PI intensity in each image containing in average 100 cells was done after thresholding and subtraction of background using Fiji (Schindelin et al., 2012) on at least three images per experiment and repeated three times, independently.

### Live-cell microscopy

For live-cell TIRF microscopy cells were seeded on 1.5 high tolerance glass coverslips (Warner Instruments) and for spinning disc microscopy on coverslips or in a CellAsic microfluidics plate (Merck Millipore). Cell seeding was done 6 h after transfection in a density to reach 50–70% confluency the day after. Live-cell microscopy was performed at 5% CO_2_ and 37°C. Spinning disc confocal live-cell microscopy movies were acquired using a 63× objective lens (Plan-Apochromat 1.40 Oil DIC M27) in a Cell Observer Spinning Disc Confocal Microscope system (ANDOR iXon Ultra) (Zeiss) and TIRF microscopy using a 100× TIRF oil (NA 1.46) DIC lens controlled by the ZEN Software. All live-cell microscopy experiments were performed in phenol red free DMEM containing 1.8 mM CaCl_2_ supplemented with 15 mM HEPES and 1 mM sodium pyruvate. The LLO concentration used was titrated for each independent experiment to give optimal amount of damage. All images were prepared using Fiji and Photoshop CS5 (Adobe), and movies were made in Fiji. Time 0 represents the start of acquisition if not stated otherwise.

### Co-localisation assays

Flp-In TRex cells induced with doxycycline and transfected with ANXA6-mCherry were washed 2× in serum free DMEM and placed in the microscope in 500 μl medium. LLO was added to the cells in 300 μl medium and live-cell confocal spinning disc movies were acquired at 1 fps for 5 min. The LLO concentration was titrated for each independent experiment to give optimal amount of damage. Alternatively, the experiment was done using microfluidics plates and continuous imaging. For microfluidics the cells were first washed for 5 min at 4 psi with serum free DMEM before being flushed with LLO toxin at 4 psi for 10 min in serum free DMEM. The localisation of ANXA6-mCherry and endocytic proteins was analysed using Fiji. ANXA6-mCherry structures were divided into three subcategories and analysed in Fiji using a 4-pixel-wide (0.85 μm) line drawn from the extracellular space into the cytosol crossing the ANXA6-mCherry spot. The peak intensities of both channels were measured at the moment of ANXA6-mCherry structure appearance as well as 10 s before and 10, 30 and 60 s after appearance. Overlapping intensity peaks in both channels were interpreted as co-localisation, peaks within 1 μm was categorised as proximal. For category 2, where the highest ANXA6-mCherry intensity was outside of the cell in thin protrusions the localisation of endocytic proteins was investigated both at the base of the protrusion and at the high intensity area in the protrusion. Three independent experiments were performed for each condition and all observed events were pooled and presented in a bar plot. Analysis of Pearson’s coefficient and Manders coefficient over time was performed using Imaris Imaris (Bitplane). Three cells from three independent experiments were analysed for each condition.

### Quantification of fluorescent intensity and the number of spots over time

Flp-In TRex cells induced with doxycycline and transfected with ANXA6-mCherry were washed 2× in serum free DMEM and placed in the microscope in 500 μl medium. Live-cell TIRF microscopy imaging of ANXA6-mCherry was performed at every 3 s for 5 min before and 30 s after addition of LLO in 300 μl medium. The LLO concentration was titrated for each independent experiment. The number of spots over time in each movie with visible ANXA6-mCherry recruitment to the plasma membrane was quantified using the spots function in Imaris (Bitplane). Assemblies from TIRF movies above 0.5 μm for caveolin1, EHD2 and GRAF1 and 1.0 μm for SNX9 were segmented as spots. The quality threshold of the spots was adjusted manually to cover all visible structures. The experiment was repeated three times for each condition by acquiring 1–4 movies containing between 1–5 cells. For analysis of the number of GRAF1 spots in the spinning disc movies using Imaris (Bitplane), the spot segmentation was set at 0.8 μm. Analysis of Pearson’s and Manders coefficients were performed using Imaris software.

### Fluo-3 and FM4-64 analysis

For analysis of Ca2+ influx, cells were seeded on glass coverslips and transfected with ANXA6-mCherry one day before the experiment. The cells were then incubated for 30 min with 1 μM Fluo-3 AM in DMEM supplemented with 10% FBS, washed and incubated for another 30 min. Cells were put in DMEM containing 1.8 mM CaCl_2_ without FBS and imaged for 1 min before addition of LLO using live-cell confocal spinning disc microscopy at 1 fps. 30 s after addition of LLO the cells were imaged for 5 min at 1 fps and the intensities of cytosolic Fluo-3 and emerging ANXA6-mCherry spots were measured using the plot z-axis profile function in Fiji. For analysis of membrane damage using FM4-64, cells were seeded on coverslips and transfected with ANXA6-BFP 16 h before the experiment. 2.5 μg/ml FM4-64 and LLO toxin was added to the cells at the same time. Imaging by spinning disc confocal live-cell microscopy was started 30 s after addition of LLO and FM4-64 at 1 fps.

### Manipulation of intracellular Ca^2+^ levels

For quantification of GRAF1 spots after drug treatment in fixed cells, 1.5×10^5^ Hela Flp-In TRex cells expressing GFP-GRAF1 were seeded onto coverslips in 24-well plates. The cells expressing GFP-GRAF1 were incubated for 30 min with either 10 μM Ionomycin or 50 μM Bapta-AM, fixated and imaged using confocal microscopy. GFP-GRAF1 spots were quantified using Imaris or ImageJ to analyse 3D micrographs and maximum-projected confocal z-stacks, respectively. GRAF1 structures above 0.5 μm were segmented as spots and the intensity threshold was adjusted manually to cover all visible GRAF1 structures. In total, 100 cells per condition were analysed in three separate experiments. Confocal z-stacks of fixed cells were captured using a 60× lens (Plan-Apochromat 1.40 Oil DIC 0.17) A1 R Laser Scanning Confocal Microscope system (Nikon Instruments) under control of the NIS-Elements Microscope Imaging Software.

### Statistical analysis

Statistical data analysis was performed with Prism (GraphPad Software) with the indicated sample size and number of independent experiments. All bar plots are presented as means±s.d., *P≤0.05, **P≤0.01 and ***P≤0.001 and non-significant (ns).

## Supplementary Material

Supplementary information
