# Supplementary material for: Plasma membrane damage caused by listeriolysin O is not repaired through endocytosis of the membrane pore
Source: Biol Open. 2018 Sep 25;7(10):bio035287. doi: 10.1242/bio.035287 (PMC6215411; doi:10.1242/bio.035287)
Supplement: Supplementary information [file biolopen-7-035287-s1.pdf]

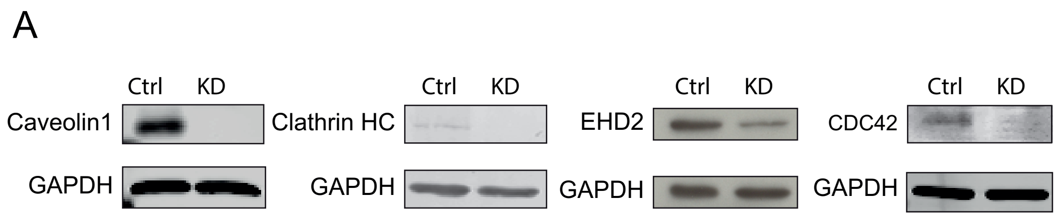

Fig. S1. Titration of LLO concentrations on HeLa cells. (A) Representative immunoblots of HeLa cells treated with different siRNAs as indicated. GAPDH or Clathrin HC were used as loading controls. These siRNAs have been previously verified and quantified in our laboratory; caveolin1 and clathrin (Mohan et al., 2015), GRAF1 (Doherty et al., 2011; Holst et al., 2017; Lundmark et al., 2008; Vidal-Quadras et al., 2017) and cdc42 (Francis et al., 2015; Vidal-Quadras et al., 2017).

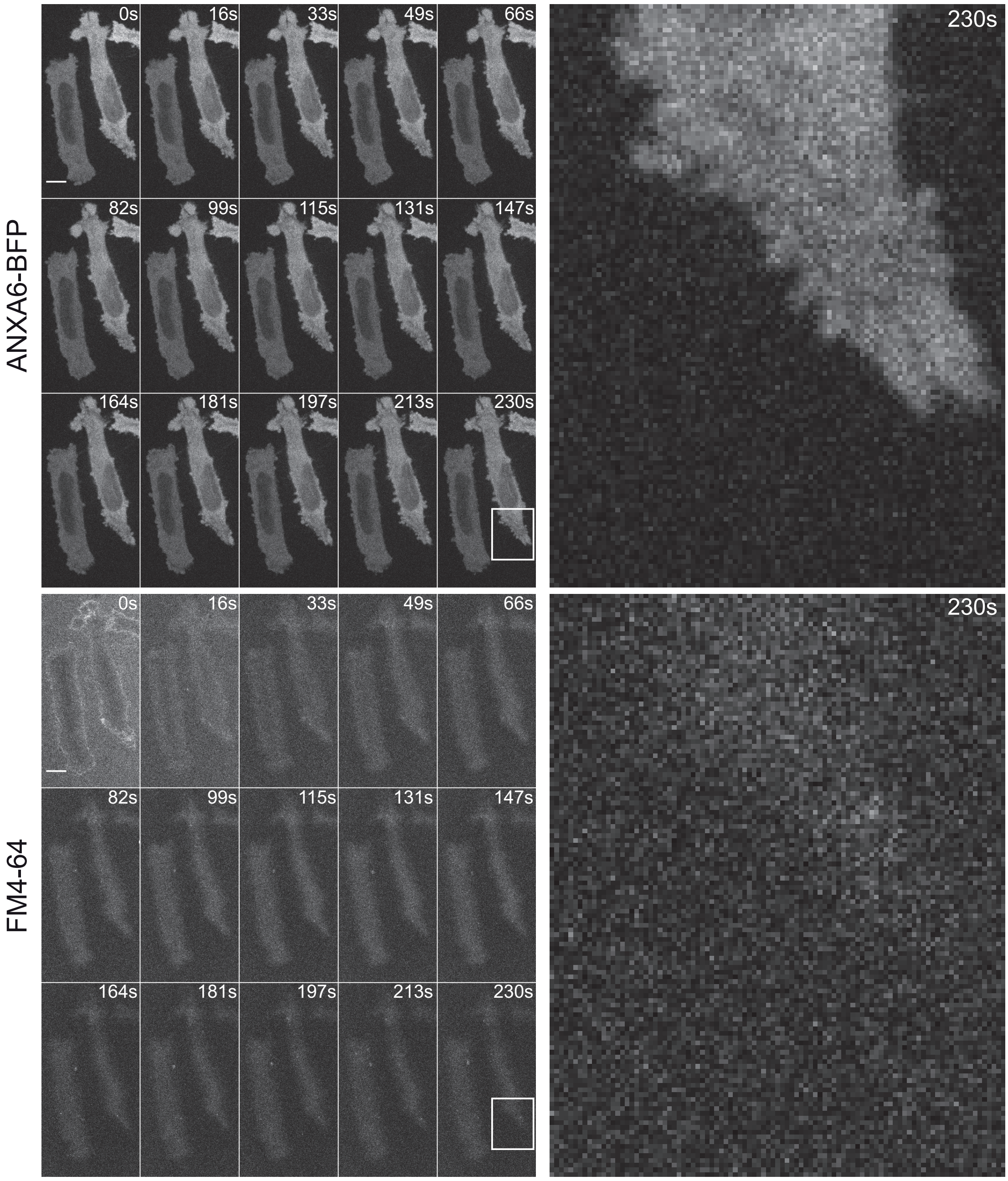

Fig. S2. FM4-64 does not label the membrane of cells with an intact plasma membrane. Live-cell confocal spinning disc time series of ANXA6-BFP (top) transfected HeLa Flp-In TRex cells treated with control buffer and FM4-64 (bottom). To the right, insets of the white box form the last frame of each channel. Time in seconds and scale bars 10 µm.

Supplementary Figure 3

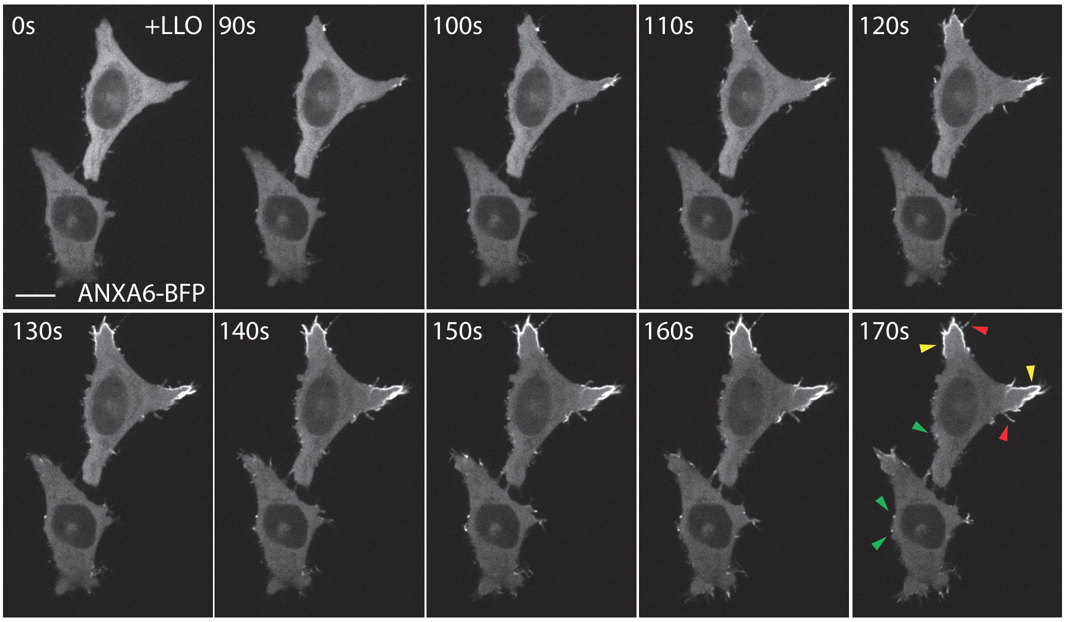

Fig. S3. Annexin A6 assembles in punctae, protruding spikes and larger surface areas in response to LLO. Live-cell confocal spinning disc time series of ANXA6-BFP (top) transfected HeLa Flp-In TRex cells treated with LLO. Arrow heads indicate punctae (green), protruding spikes (red) and larger surface areas (yellow). Time in seconds and scale bars 10 µm.

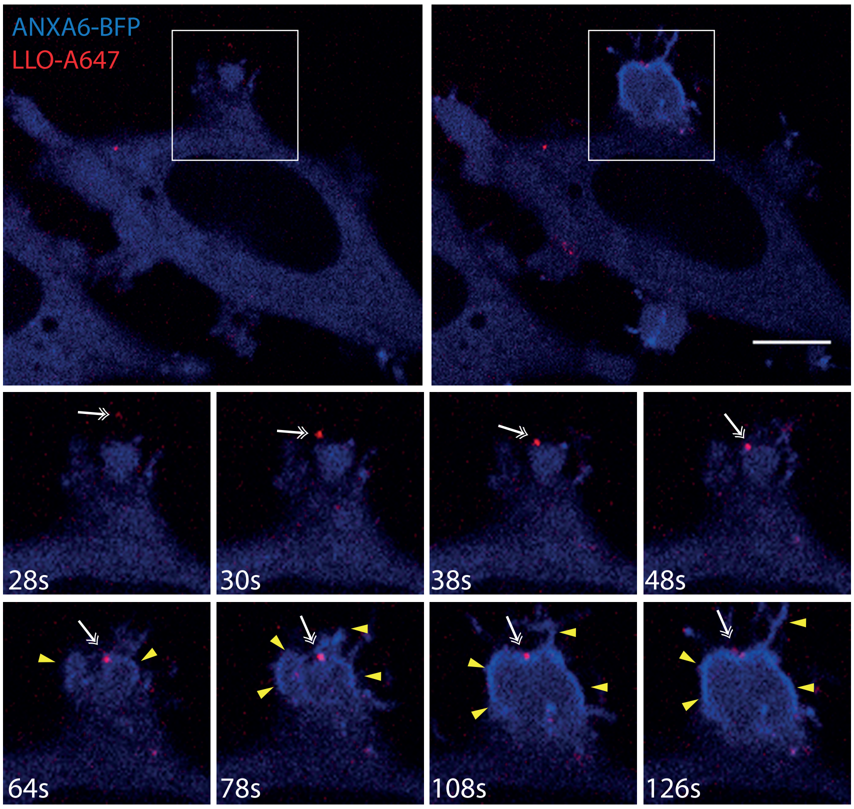

Fig. S4. LLO-A647 triggers a specific Annexin A6 response. Fluorescent micrographs from live-cell confocal spinning disc imaging taken every other second of cells transfected with ANXA6-BFP (blue) and treated with LLO-A647 (red). Insets show time series in magnification of the area indicated by a white rectangle. White double arrow indicates a LLO-A647 punctae and yellow arrowheads indicate the annexin response. Time in seconds and scale bar 10 µm.

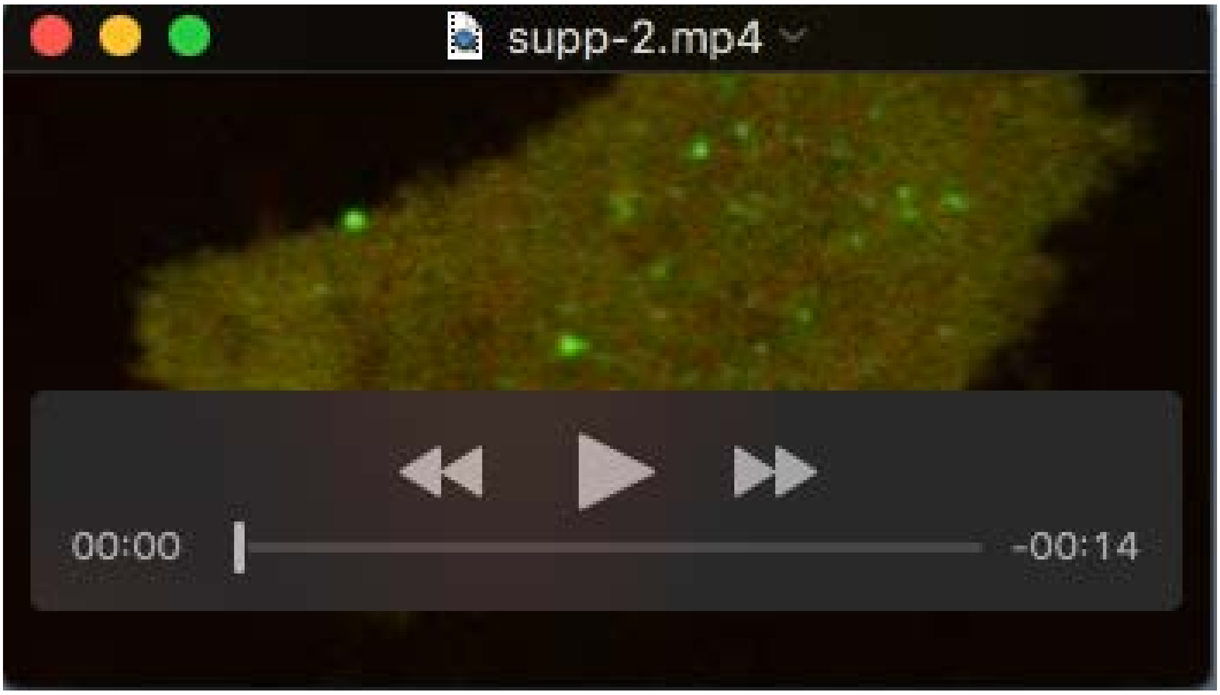

Movie 1. Confocal spinning disc movie taken at 1 fps corresponding to figure 3A of ANXA6-mCherry (red) transfected GFP-SNX9 (green) Flp-In TRex cells treated with LLO. Time 5 min shown in mm:ss. Scale bar 2 µm.

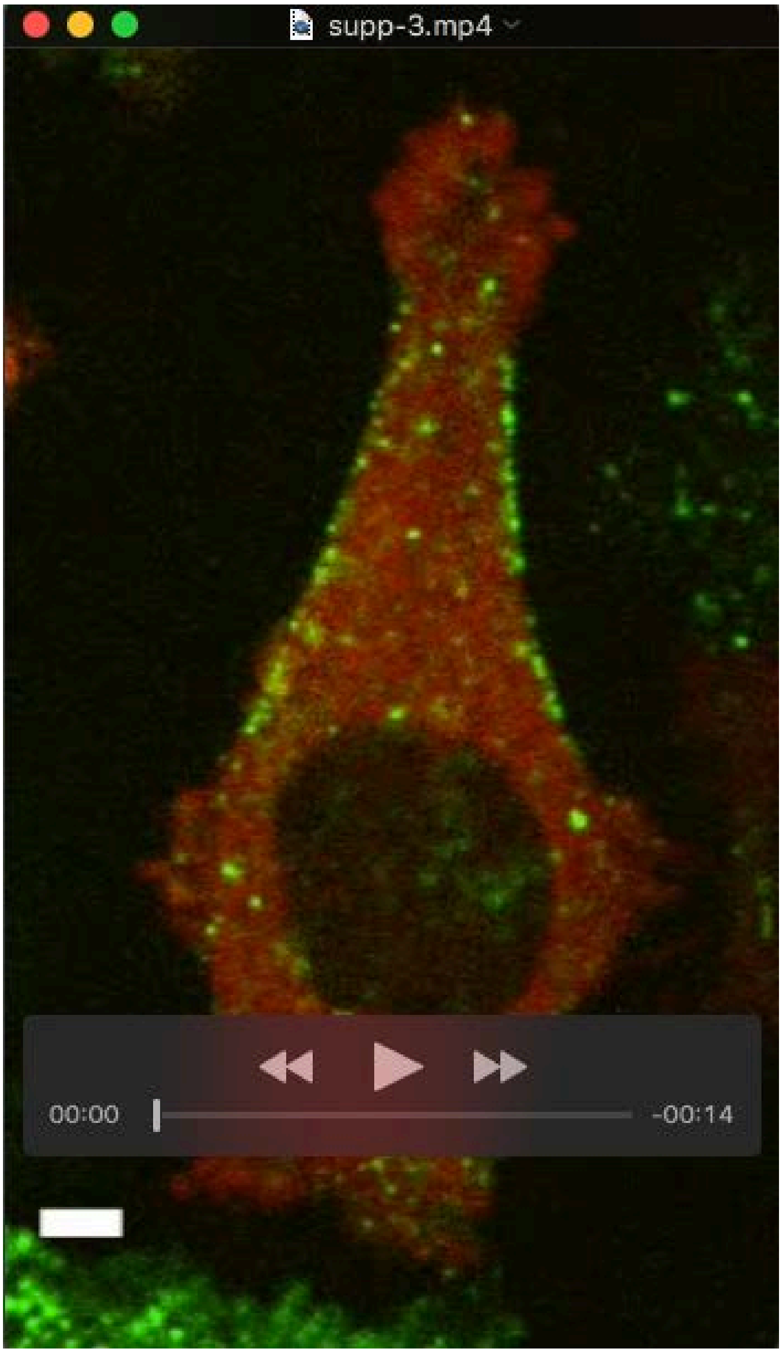

Movie 2. Confocal spinning disc movie corresponding to figure 3A of ANXA6-mCherry (red) transfected caveolin1-GFP (green) Flp-In TRex cells treated with LLO. Time 5 min shown in mm:ss. Scale bar 2 µm.

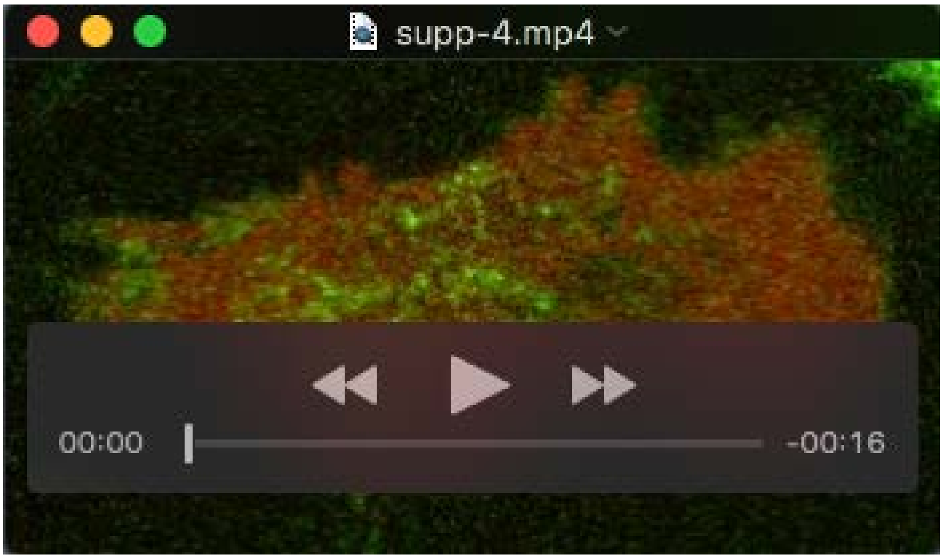

Movie 3. Confocal spinning disc movie taken at 1 fps corresponding to figure 3A of ANXA6-mCherry (red) transfected EHD2-GFP (green) Flp-In TRex cells treated with LLO. Time 5 min shown in mm:ss. Scale bar 2 µm.

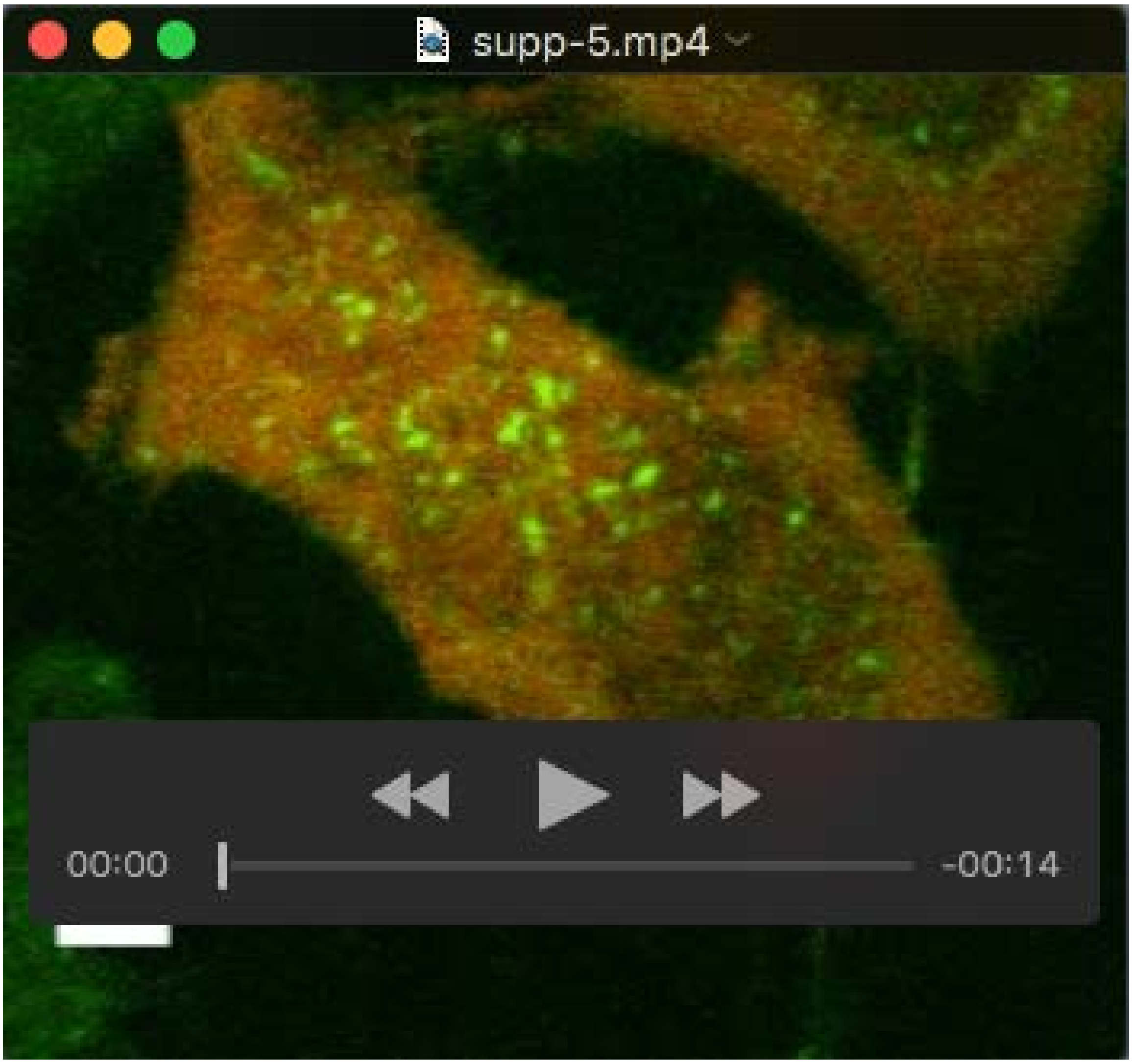

Movie 4. Confocal spinning disc movie taken at 1 fps corresponding to figure 3A of ANXA6-mCherry (red) transfected GRAF1-GFP (green) Flp-In TRex cells treated with LLO. Time 5 min shown in mm:ss. Scale bar 2 µm.

Biology Open • Supplementary information

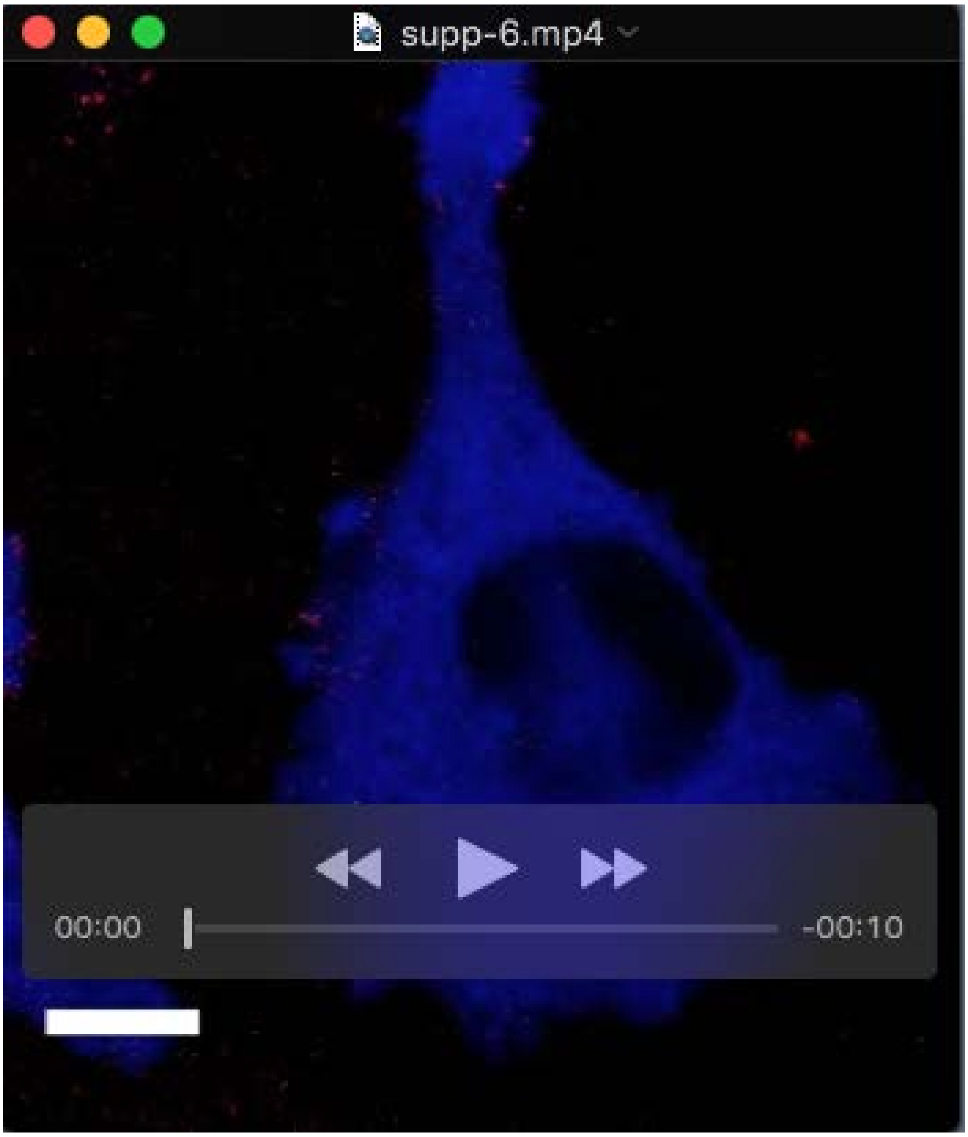

Movie 5. Confocal spinning disc movie taken at 2 s per frame corresponding to figure 4A of ANXA6-BFP (blue) transfected Flp-In Trex cells treated with LLO-A647 (red). Time 5 min shown in mm:ss. Scale bar 10 µm.

Biology Open • Supplementary information

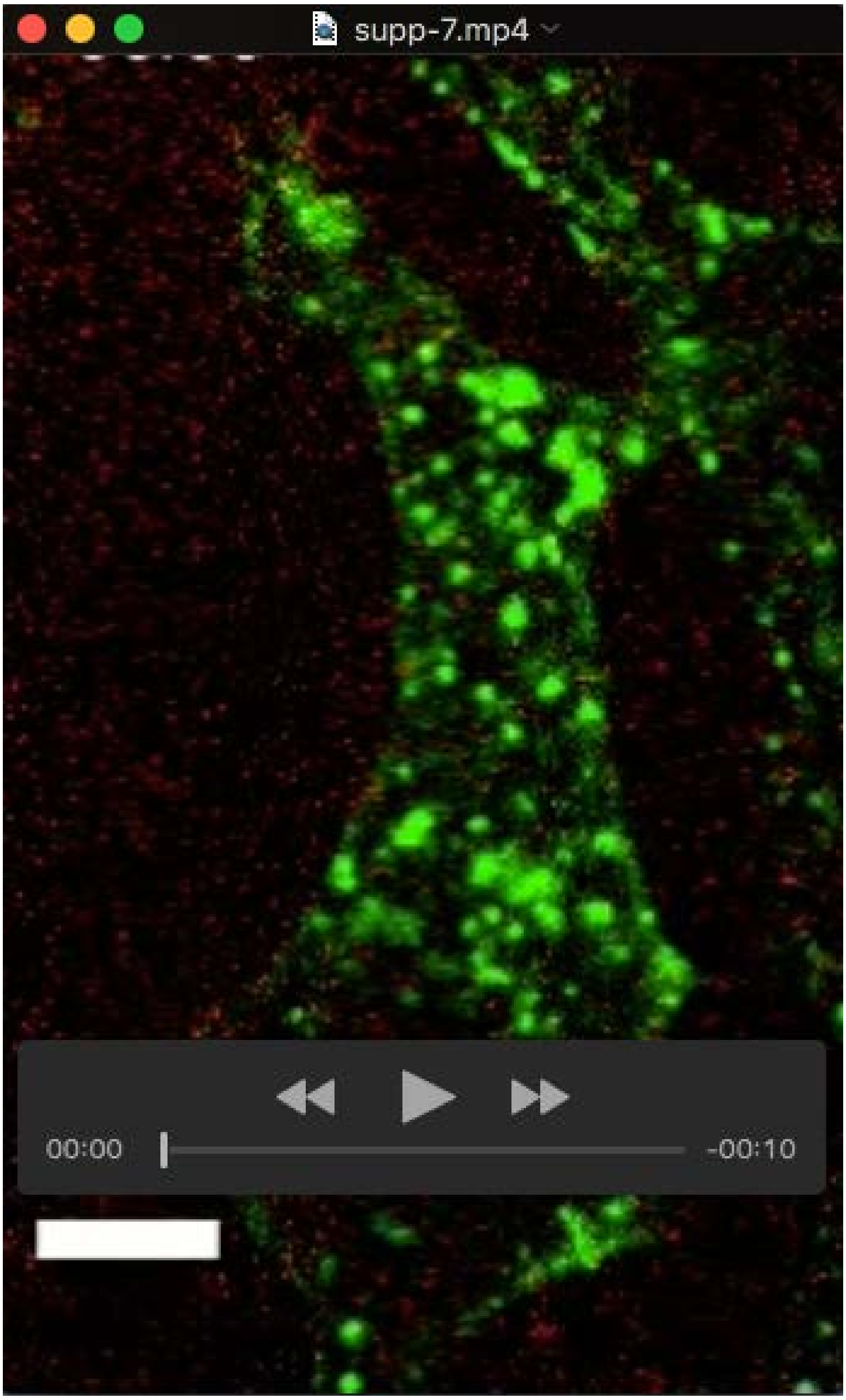

Movie 6. Confocal spinning disc movie taken at 2 s per frame corresponding to figure 4B of caveolin1-GFP Flp-In Trex cells treated with LLO-A647 (red). Time 5 min shown in shown in mm:ss. Scale bar 8 µm.

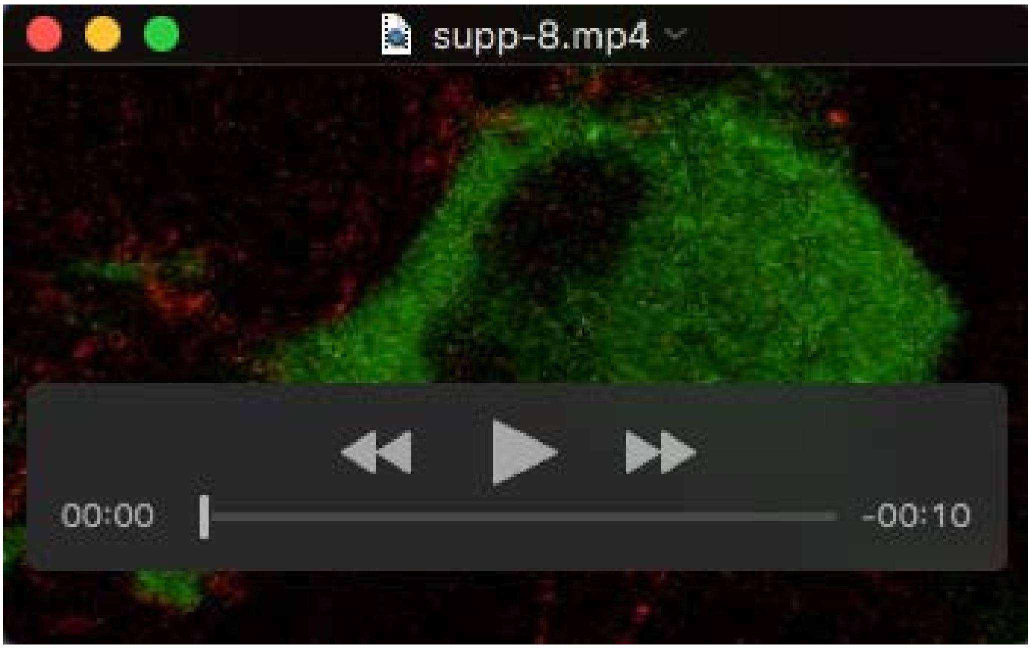

Movie 7. Confocal spinning disc movie taken at 2 s per frame corresponding to figure 4B of GRAF1-Flp-In Trex cells treated with LLO-A647 (red). Time 5 min shown in mm:ss. Scale bar 8 μm.

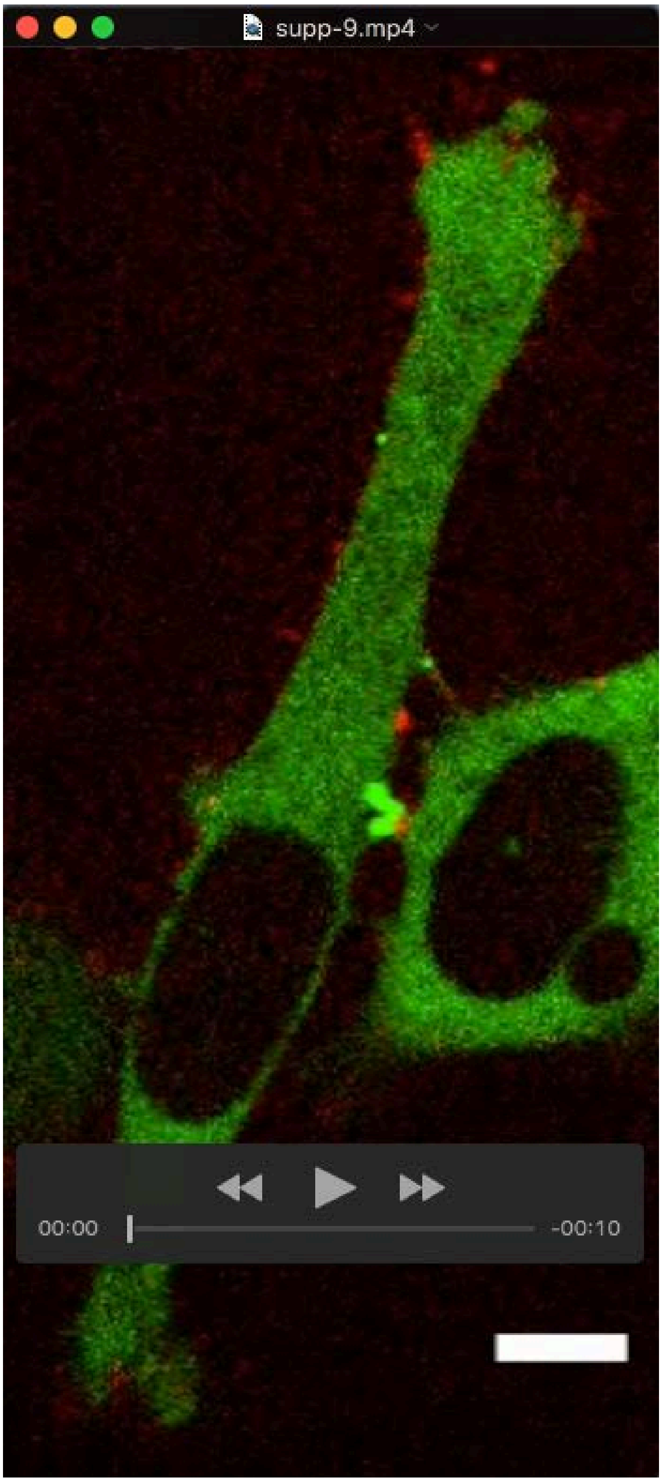

Movie 8. Confocal spinning disc movie taken at 2 s per frame corresponding to figure 4B of GFP-SNX9 Flp-In Trex cells treated with LLO-A647 (red). Time 5 min shown in mm:ss. Scale bar 8 µm.

Biology Open • Supplementary information

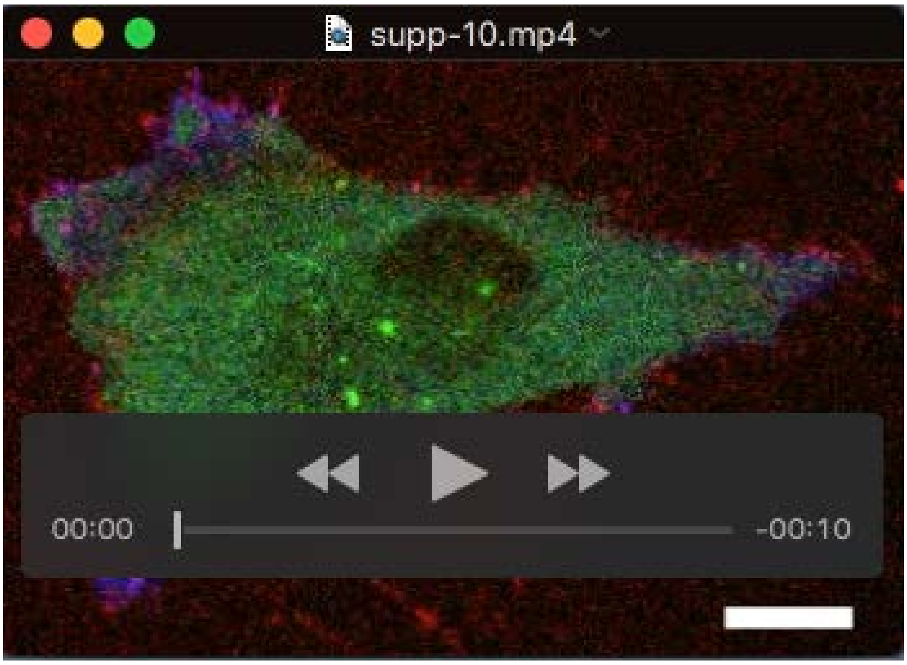

Movie 9. Confocal spinning disc movie taken at 2 s per frame corresponding to figure 4E of ANXA6-BFP (blue) transfected GRAF1-GFP (green) Flp-In TRex cells treated with LLO-A647. Time 5 min shown in mm:ss. Scale bar 10 µm.

Biology Open • Supplementary information

Supplemental References

Doherty, G.J., M.K. Ahlund, M.T. Howes, B. Moren, R.G. Parton, H.T. McMahon, and R. Lundmark. 2011. The endocytic protein GRAF1 is directed to cell-matrix adhesion sites and regulates cell spreading. *Molecular biology of the cell*. 22:4380-4389.

Francis, M.K., M.R. Holst, M. Vidal-Quadras, S. Henriksson, R. Santarella-Mellwig, L. Sandblad, and R. Lundmark. 2015. Endocytic membrane turnover at the leading edge is driven by a transient interaction between Cdc42 and GRAF1. *J Cell Sci*. 128:4183-4195.

Holst, M.R., M. Vidal-Quadras, E. Larsson, J. Song, M. Hubert, J. Blomberg, M. Lundborg, M. Landstrom, and R. Lundmark. 2017. Clathrin-Independent Endocytosis Suppresses Cancer Cell Blebbing and Invasion. *Cell Rep*. 20:1893-1905.

Lundmark, R., G.J. Doherty, M.T. Howes, K. Cortese, Y. Vallis, R.G. Parton, and H.T. McMahon. 2008. The GTPase-activating protein GRAF1 regulates the CLIC/GEEC endocytic pathway. *Curr Biol*. 18:1802-1808.

Mohan, J., B. Moren, E. Larsson, M.R. Holst, and R. Lundmark. 2015. Cavin3 interacts with cavin1 and caveolin1 to increase surface dynamics of caveolae. *J Cell Sci*. 128:979-991.

Vidal-Quadras, M., M.R. Holst, M.K. Francis, E. Larsson, M. Hachimi, W.L. Yau, J. Peranen, F. Martin-Belmonte, and R. Lundmark. 2017. Endocytic turnover of Rab8 controls cell polarization. *J Cell Sci*. 130:1147-1157.

Biology Open • Supplementary information
